# Antibiotics Coupled with Photothermal Therapy for the Enhanced Killing of Bacteria

**DOI:** 10.51847/nplvoycg9u

**Published:** 2023-09-26

**Authors:** Amanda Jalihal, Armin Mortazi, Mavis Forson, Mujeebat Bashiru, Thuy Le, Adeniyi Oyebade, Noureen Siraj

**Affiliations:** Department of Chemistry, University of Arkansas at Little Rock, 2801 S. University Ave, Little Rock, AR 72204, USA.

**Keywords:** Ionic material, Photothermal therapy, Combination therapy, Antibiotic resistance

## Abstract

In this study, the application of ionic materials as a combination antibiotic drug was investigated. The fluoroquinolone, Norfloxacin, was converted into the ionic form and combined with the cationic dye, IR780^+^, using an ion-exchange reaction. The resulting ionic combination drug possesses two killing mechanisms in one compound. The antibiotic chemical mechanism along with the photothermal mechanism that was acquired by adding IR780 to the compound led to the development of a combination antibiotic drug. This ionic combination drug consisting of Norfloxacin anion and IR780 cation is easily dispersed in water using sonication waves. The parent compounds and ionic combination drug, dissolved in organic solvent and dispersed in water, were characterized, and the photophysical properties were studied in detail. It was discovered that the aqueous ionic combination drugs exhibited significant changes in absorbance and photoluminescent properties. In aqueous media, the dispersed ionic combination drug exhibited a very broad absorbance with an additional peak around 1000 nm which is advantageous in photothermal. A significant decrease in the quantum yield along with enhanced non-radiative rate constant was observed for the combination drug in the aqueous. The photothermal mechanism is present in both the parent IR780 dye and the ionic combination drug. The ionic combination drug displayed a high light-to-heat conversion efficiency and temperature increase similar to the parent dye. The combination of both killing mechanisms in the ionic combination drug resulted in enhanced antibacterial activity against *Escherichia coli* as compared to the parent Norfloxacin and IR780-I individually.

## Introduction

One of the most daunting issues of our time is the emergence of “superbugs” caused by antibiotic resistance in bacteria (Liu *et al*., 2018). Resistance occurs naturally in bacteria when a mechanism of defense develops against antibiotics through natural selection (Centers for Disease Control, 2019). Over- and misuse of antibiotics are the leading causes of the development of drug resistance (Qing *et al*., 2019). Examples of antibiotic resistance include enzymatic modification to the antibiotic, decreased membrane permeability, antibiotic removal from the cell via efflux pumps, etc. (Wang *et al*., 2022). To date, several options have emerged to combat such pathogens including silver ions (Roy *et al*., 2019), polymers containing multiple treatments (Dizman *et al*., 2005), bacteriophage-antibiotic combinations (Li *et al*., 2021; Van Nieuwenhuyse *et al*., 2022), and smart nanoparticles (Qing *et al*., 2019). These studies indicate that resistant bacteria can be eliminated with little thread of defense development through the combination of physical and chemical killing mechanisms.

Extermination of bacteria by a physical mechanism results in an irreversibly denatured or disrupted bacterial cell. High temperatures, pH changes, and radiation are examples of physical mechanisms to annihilate bacteria (Amit *et al*, 2017). Whereas, bacteria death through a chemical mechanism involves interference with the cell’s metabolic pathways usually via antibiotics or antiseptics (Blondeau, 2004). Recently, dual mechanism treatments have been utilized as an effective strategy to exterminate bacteria. This type of treatment induces a synergistic effect to effectively eliminate bacterial infections. Dual mechanism therapies can reduce the risk of resistance development, enhance bacterial suppression, increase antibiotic activity in biofilms, and increase infection clearance rates (Chanda & Rakholiya, 2011; Sullivan *et al*., 2020; Li *et al*., 2021; Zhu *et al*., 2021). Previous studies combine physical and chemical mechanisms through covalent bonding (Zhao *et al*., 2019) or polymeric backbone (Chiang *et al*., 2015; Liu *et al*., 2018). While the combination strategies significantly increased the antibiotic activity of the drug, these combination drugs required complex and lengthy synthesis (Chiang *et al*., 2015; Liu *et al*., 2018; Van Nieuwenhuyse *et al*., 2022). Thus, it is essential to develop new economical and simple synthesis strategies that can produce a combination drug with high yield.

An interesting physical killing mechanism that is getting tremendous attention is the photoactive treatment known as photothermal therapy (PTT). In PTT, light energy is transformed into heat and increases the temperature of the surrounding tissues (Gharatape *et al*., 2016). Normally, temperatures ≥50°C will irreversibly denature bacterial protein and enzymes, ultimately leading to cell death (Chiang *et al*., 2015). Implementation of PTT as an antibacterial treatment should eliminate the possibility of resistance development because the cell is physically destroyed. Additionally, PTT requires electromagnetic radiation that is absorbed by the photosensitizer to initiate the heating mechanism which allows for a targeted approach. In the absence of light, heat is not generated by photosensitizer thus reducing the risk of unwanted toxicity (Yan *et al*., 2020). However, the requirement of light to induce the PTT mechanism can also be a limitation since light is absorbed by tissues as well. Near-infrared (NIR) light (600–900 nm wavelength) penetrates farther into tissues as compared to visible and ultraviolet light (Jiao *et al*., 2018). In order to treat bacterial infections in deeper tissues, a NIR-absorbing PTT agent that absorbs at longer wavelengths of electromagnetic radiation is advantageous.

The novel ionic material synthesis is a potential solution for the development of a dual-mechanism antibiotic. Previously, ionic materials have been developed as a combination drug for use in cancer treatment by combining chemotherapeutic and photoactive moieties (Macchi *et al*., 2021). Using this ionic material approach both physical and chemical mechanisms are combined electrostatistically in a single compound to eradicate bacteria (Jordan *et al*., 2014; Siraj *et al*., 2014; Warner *et al*., 2014). This class of ionic combination drug (ICD) has potential application as an effective antibacterial treatment. The innovative approach entails a double displacement ion-exchange reaction between antibiotic and NIR dye (Jordan *et al*., 2014; Siraj *et al*., 2014; Warner *et al*., 2014). The resulting ICD is hydrophobic but can be dispersed in aqueous media under sonication waves (Xie *et al*., 2011; Tiwari *et al*., 2018; Roy *et al*., 2019) to be used practically in biological environments.

Herein, an ICD is proposed for use as an effective antibacterial treatment. The fluoroquinolone, Norfloxacin (NOR^−^), and near-infrared (NIR) dye (IR780-I) were selected as the chemical and physical killing moieties, respectively. NOR^−^ has shown broad-spectrum activity against bacteria and causes cell death through inhibition of DNA gyrase (Norrby *et al*., 1983). IR780^+^ has been previously reported to possess the PTT mechanism (Kuang *et al*., 2017; Chang *et al*., 2019). By combining these two moieties (NOR^−^ and IR780^+^ as an ICD, dual activity can be achieved promptly with fewer materials required (Kuang *et al*., 2017). By combining these distinct killing mechanisms, a synergistic effect should allow for enhanced antibacterial activity at lower concentrations.

## Materials and Methods

### Materials

IR780-I, Norfloxacin, and K12 *e. coli* cells were purchased from Sigma Aldrich (Saint Louis, MO). Petri dishes, dichloromethane (DCM), and dimethylsulfoxide (DMSO) were purchased from VWR (Radanar, PA). Nutrient agar and Mueller Hinton (MH) broth were purchased from Thermo Scientific (Waltham, MA). Distilled deionized water of 18.2 MΩ cm was obtained from an ultrapure distilled water purifier (ELGA) and used for all studies.

### ICD Synthesis

**Scheme 1.**
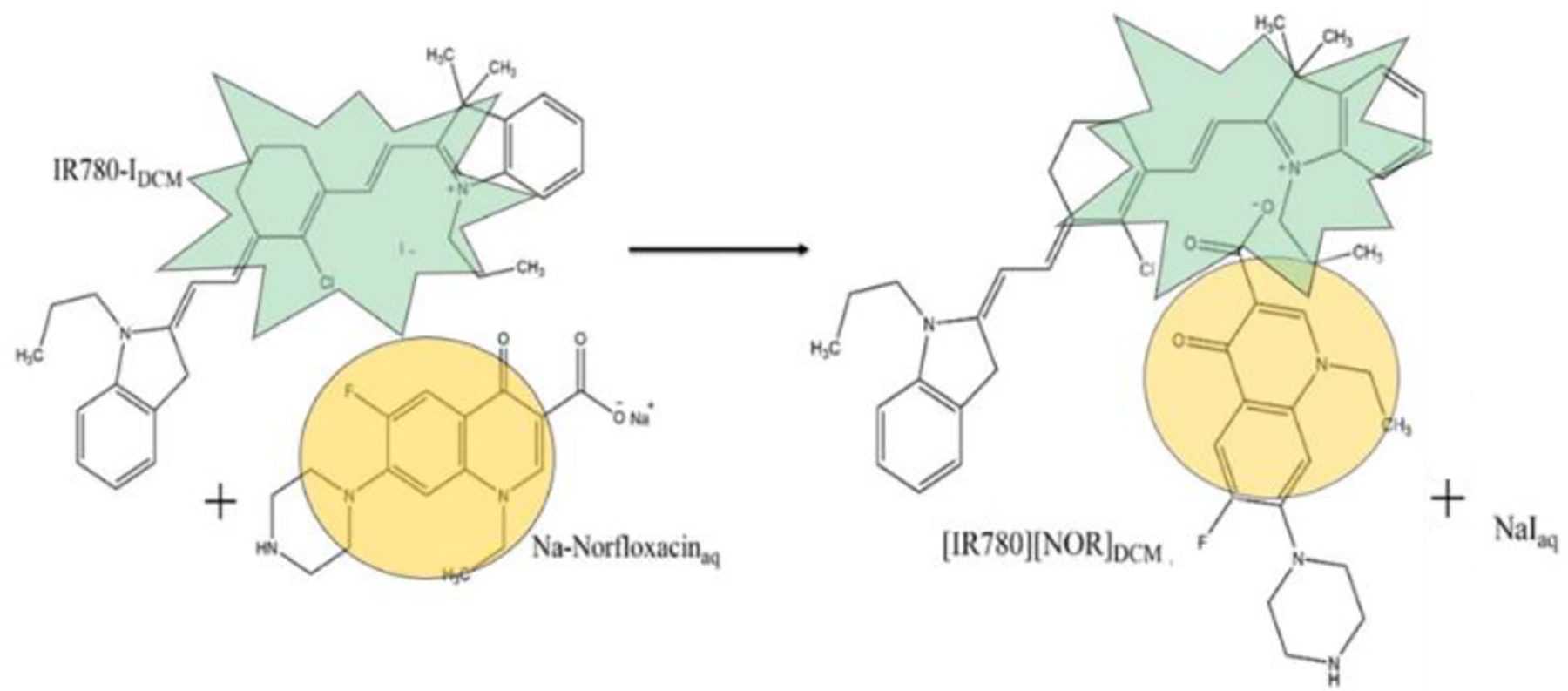
Reaction scheme of the ICD [IR780][NOR]

The ICD, [IR780][NOR], was synthesized using a previously reported method (Blondeau, 2004). First, NOR and IR780-I were prepared in a 1:1 molar ratio in the respective solvents. NOR was dissolved in water (pH 9), and IR780-I was dissolved in DCM at a volume of at least five times that of the aqueous solution to better enable an ion exchange reaction. The two solutions were combined in a single vial and stirred for 48 hours at room temperature. After the time had elapsed, the ions of interest (IR780^+^ and NOR^−^) were present in the organic layer. The spectator ions (Na^+^ and I^−^) migrated to the aqueous layer, and were easily removed with the water layer. The organic layer was washed several times to remove any residual spectator ions. To ensure the complete removal of iodide ions, a silver nitrate test was performed. The organic solvent was evaporated which resulted in a pure ICD, illustrated in Scheme 1. The solid ICD was lyophilized to remove any water impurities. By using this technique, the ions of interest were combined and spectator ions were removed simultaneously. This scheme allows us to create the combination drug, [IR780][NOR] without a complicated or multistep synthesis protocol.

The ICD product was hydrophobic but must be used in an aqueous environment for bacterial studies. To solve this issue, the ICD was dispersed in water (Jalihal *et al*., 2021). First, the ICD was dissolved in a water-miscible organic solvent at a high concentration. In this work, DMSO was selected as the solvent. A small aliquot of this solution was added to a volume of water under sonication waves for 5 minutes and rested for 20 minutes. In this process, the organic solution was dispersed into the water resulting in the ICD colloid in aqueous media. In preliminary studies, 2% DMSO (v/v) was deemed to be negligibly toxic to *E. coli*. It was carefully calculated that all dispersed aqueous mixtures contained no more than 2% DMSO (v/v). Additionally, all negative controls for antibiotic experiments included 2% DMSO (v/v) to ensure antibiotic activity was not due to the organic solvent.

### Characterization

The identity of the newly synthesized ICD was confirmed using a Shimadzu IT-TOF ESI high-resolution mass spectrometer. The identity of the ICD was verified by comparing the mass-to-charge ratio of both ions to the parent molecules. Thermogravimetric analysis (TGA) was performed using a Mettler Toledo instrument to verify the thermal stability of the solid ICD. Samples were heated in air at a rate of 10 °C per minute from 25–800 °C.

### Absorbance Studies

The absorbance of the ICD and parent molecules was performed to evaluate the photophysical characteristics. Samples of parent dyes (Na-Norfloxacin, IR780-I) and ICD ([NOR][IR780]) were prepared in DMSO in order to determine the presence of both ions in the ICD. Samples of the ICD dispersed in water were prepared to mimic the environment of the treatment samples. A Cary UV-60 spectrophotometer was used to analyze absorption characteristics with a 10 mm path length, polished two-sided quartz cuvette (Starna Cells). Absorbance measurements of each sample were evaluated against an identical cell filled with the solvent of the sample.

### Photophysical Properties

The fluorescence emission of the ICD and parent molecules was recorded at their respective excitation maximum using the HORIBA Jobin Yvon Nanolog fluorimeter. A 10 mm path length, polished four-sided quartz cuvette (Starna Cells) was used for fluorescence emission measurements. All samples were measured with the same slit width, the integration time of 0.1 seconds, and right angle geometry.

The quantum yield of the ICD was calculated using the relative quantum yield method, Eq. 1.

(1) $$\Phi_{un}=\Phi_{s}\left(\frac{I_{un}}{I_{s}}\right)\left(\frac{Abs_{s}}{Abs_{un}}\right)\left(\frac{\eta_{un}}{\eta_{s}}\right)$$

Where Φ is the quantum yield of the unknown (un) and standard (s), I is the integrated intensity, Abs is the absorbance at the excitation wavelength, and η is the refractive index of the media (Fery-Forgues & Lavabre, 1999). The reported quantum yield of 8% for IR780-I was used as the standard value in the calculation of the ICD quantum yield (Chapman *et al*., 2011).

The radiative rate constant (k_r_) was calculated using Eq. 2.

(2) $${\text{k}}_{\text{r}}=2.88*10^{-9}*{\text{n}}^{2}*\frac{\int \text{I}(v)\text{d}v}{\int \text{I}(v)v^{-3}\text{d}v}\int \frac{\varepsilon (v)}{v}\text{d}v$$

Where n is the refractive index, I(ν) is the fluorescence emission intensity at wavelength ν, and ε(ν) is the molar extinction at wavelength ν.

The non-radiative rate constant (k_nr_) was calculated using Eq. 3.

(3) $$\Phi_{\text{f}}=\frac{{\text{k}}_{\text{r}}}{{\text{k}}_{\text{r}}+{\text{k}}_{\text{nr}}}$$

Where Φ_f_ is the quantum yield of the compound.

The photostability of the ICD was determined by using an excitation wavelength of 780 nm light (the lambda max of IR780) and recording the fluorescence emission intensity at a single point (810 nm) continuously for 360 seconds. Both samples were measured with an entrance and exit slit width of 4 nm and at right angle geometry.

### Light-To-Heat Conversion Efficiency

The light-to-heat conversion efficiency of aqueously dispersed [IR780][NOR] and IR780-I was designed to determine if the irradiated samples can generate enough heat to kill bacterial cells. Aqueous samples of both parent dye and ICD were irradiated with an 808 nm laser for 5 minutes. During this time, the temperature of the sample was recorded every 30 seconds. A cooling curve was also measured by recording the temperature every 30 seconds without irradiation for 5 minutes. The heating and cooling curves were assessed and the light-to-heat conversion efficiency was calculated using Eq. 4.

(4) $$\eta =\frac{hS\left(T_{max}-T_{\text{surr}}\right)-Q_{dis}}{I\left(1-10^{-A}\right)}$$

Where η is the heat efficiency, h is the heat transfer coefficient, s is the surface area of the container, and hs is obtained from Eq. 5. T_max_ is the maximum steady temperature, T_surr_ is the environmental temperature, I is the laser power, A is the absorbance of the sample, and Q_dis_ is the heat dissipation from the light absorbed by the solvent and container.

(5) $$\theta =\frac{T-T_{\text{surr}}}{T_{max}-T_{\text{surr}}}$$

*θ* is a dimensionless parameter that was used in Eq. 6 to find the time constant τs.

(6) $$t=-\tau_{s}\text{ln}(\theta )$$

Where t is time. The heat transfer coefficient hs is calculated from Eq. 7.

(7) $$hs=\frac{m_{D}D}{\tau_{s}}$$

where m_D_ is the mass of the solution and C is the specific heat (Macchi *et al*., 2022).

### Percent Inhibition Studies

Bacterial cultures were grown by inoculating one *E. coli* colony in MH broth. The solution was left to grow overnight to produce a concentrated stock of bacteria used in the subsequent studies.

To investigate the activity of the ICD, percent inhibition studies were performed with and without laser irradiation. Irradiated and non-irradiated trials were prepared similarly. For the non-irradiated specimen, Na-NOR, IR780-I, and [IR780][NOR] were prepared in MH broth and diluted to concentrations 0.3, 0.6, 1.25, 2.5, and 10 μM in a 96-well plate. All wells containing treatment, positive control (10% H_2_O_2_), and negative control (2% DMSO) were inoculated with E. coli and incubated at 37°C for 24 hours. The absorbance of all samples was measured at 600 nm after incubation in a Synergy microplate reader to determine the bacterial growth. The relative number of bacteria in each treatment well was compared with the positive and negative control to quantify the percent inhibition of bacterial growth. This sample preparation process is identical to the irradiated specimen with two modifications. First, the samples are prepared in alternating wells to prevent excessive contact with the laser. Next, each well is exposed to an 808 nm laser light for 5 minutes prior to the final incubation. The percent inhibition is calculated according to Eq. 8.

(8) $$\text{percent inhibition}(\%)=\frac{Abs_{neg}-Abs_{\text{treatment}}}{Abs_{neg}}*100$$

Where Abs_neg_ and Abs_treatment_ are the absorbances of the negative control and treatment at 600 nm, respectively (Ebadzadsahrai *et al*., 2020).

## Results and Discussion

### ICD Characterization

**Figure 1.**
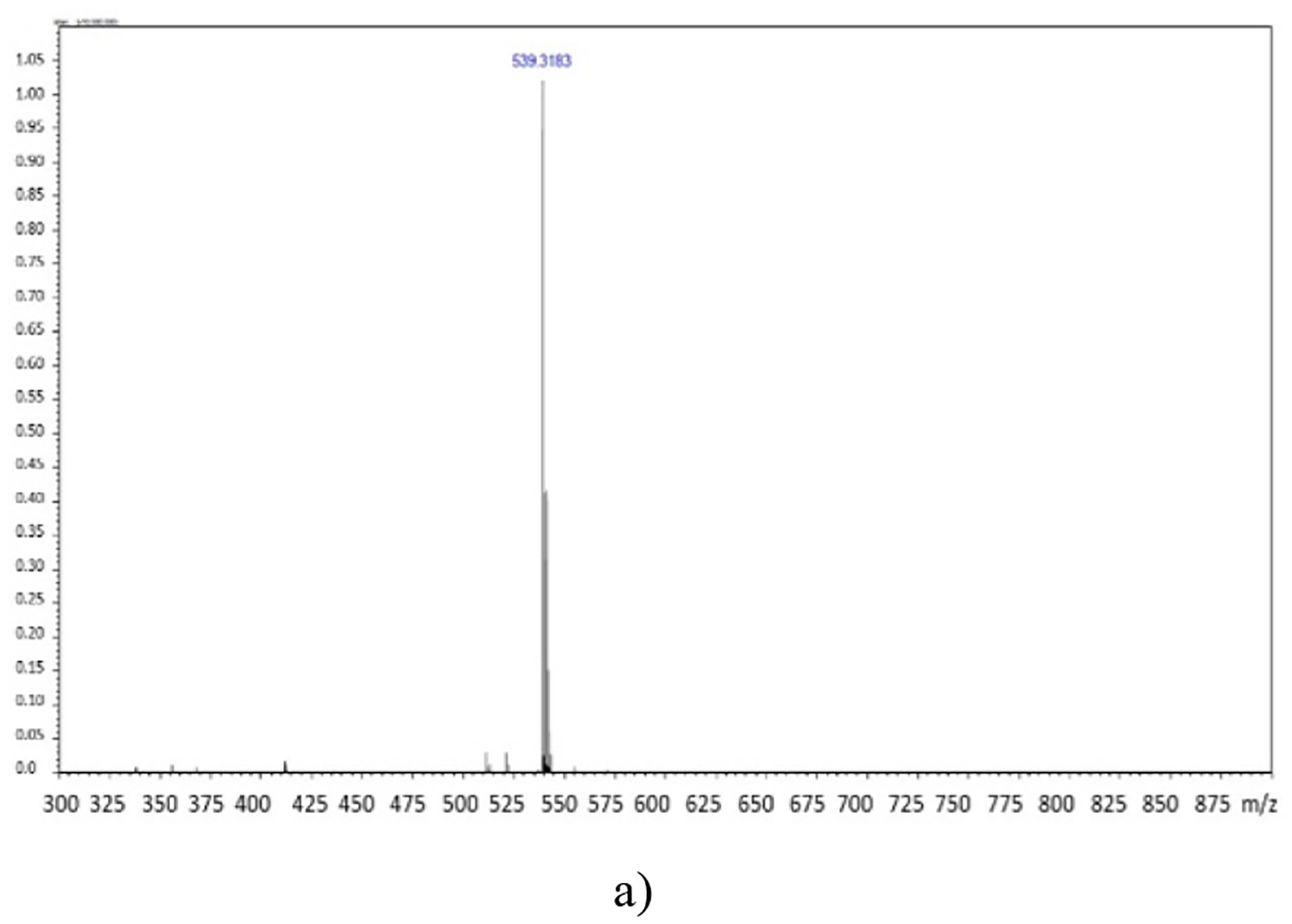

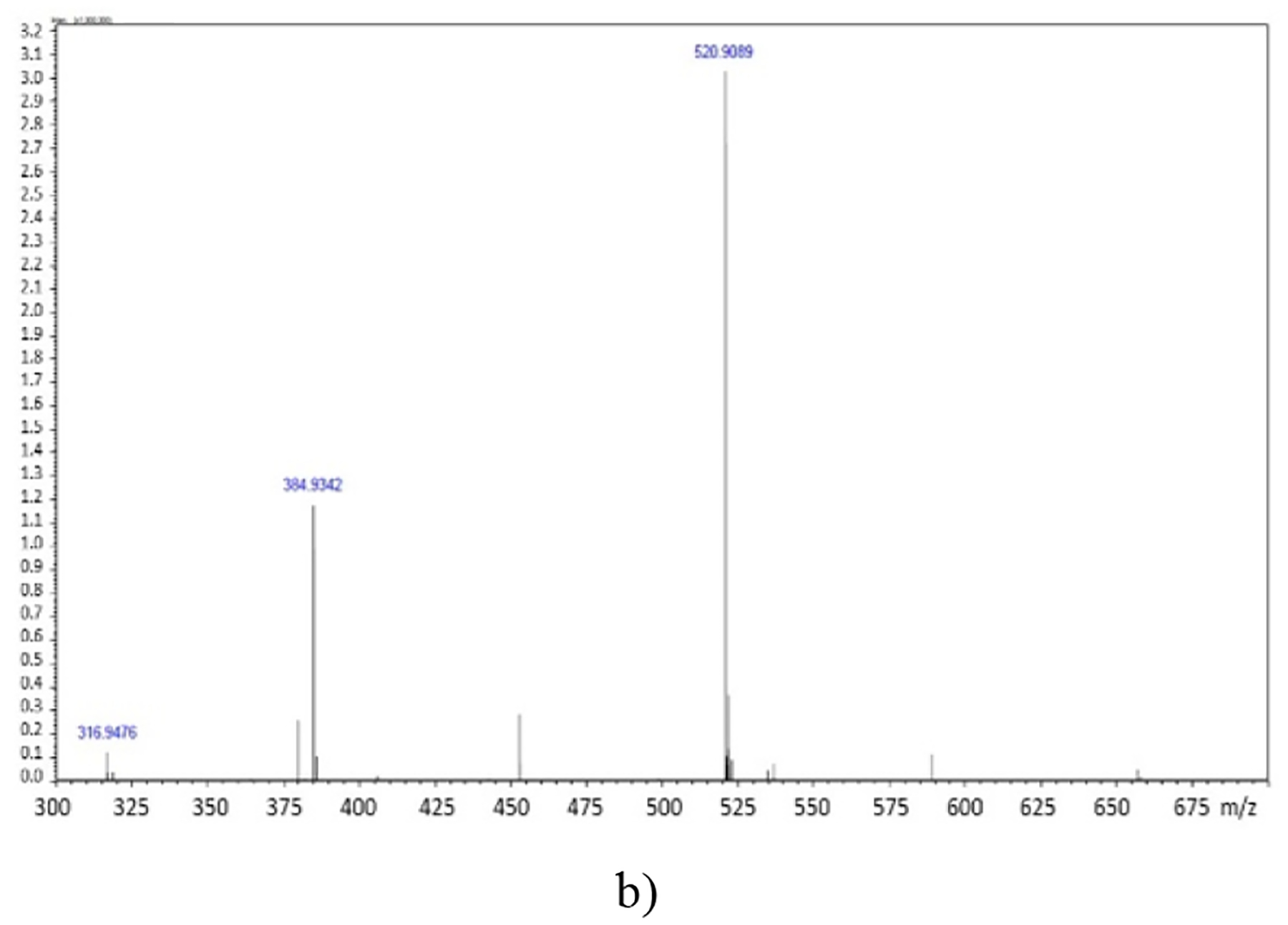
Mass spectra for [IR780][NOR] in positive (a) and negative (b) ion mode

In this work, an innovative ion exchange approach was utilized to synthesize the ICD (Jordan *et al*., 2014; Siraj *et al*., 2014; Warner *et al*., 2014) to treat bacteria effectively. Through the simple, concise method, the ICD containing IR780 and NOR was produced ([IR780][NOR]). The presence of both ions in the ICD was confirmed by mass spectrometry (Figures 1a and 1b). The observed peaks in positive and negative ion modes were consistent with the calculated molar masses for IR780^+^ (540.22 g/mol) and NOR^−^ (318.32 g/mol) at 539.3183 and 316.9476, respectively. ICD synthesis yields a highly pure product since impurities are easily removed due to solubility differences in the biphasic system.

### Photophysical Characterization

**Figure 2.**
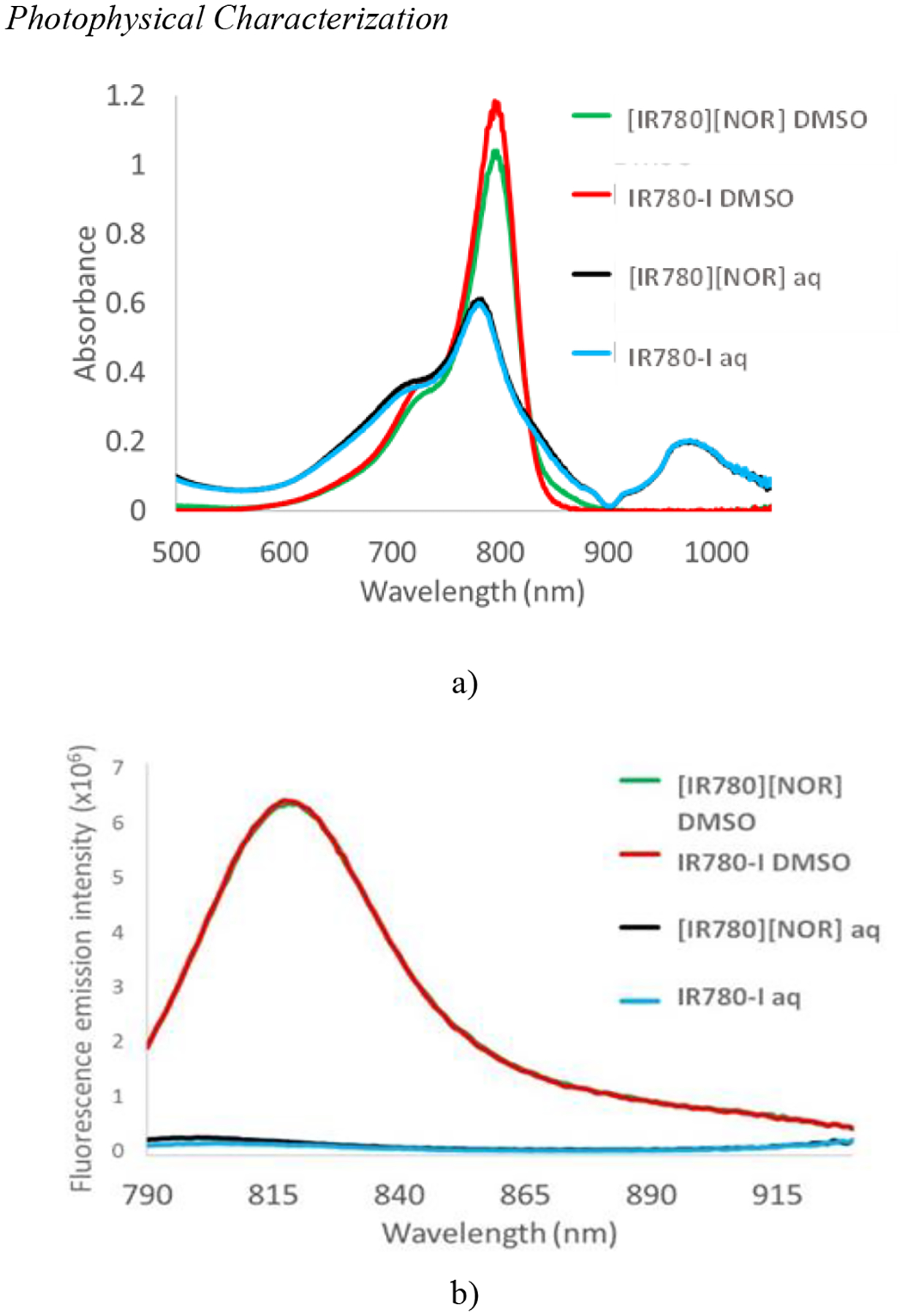
(a) Absorbance spectrum of [IR780][NOR] (DMSO), IR780-I (DMSO), [[IR780][NOR] (aq), and IR780-I (aq). (b) Emission spectrum of [IR780][NOR] (DMSO), IR780-I (DMSO), [IR780][NOR] (aq), and IR780-I (aq) excited at 784 nm.

The absorbance spectra of the parent compounds and ICD were recorded to determine if any structural modifications occurred during synthesis. The absorbance spectra of IR780-I and [IR780][NOR] dissolved in DMSO show an absorption wavelength maxima at 784 nm with a shoulder at 715 nm (Figure 2a). Both spectra display similar shapes indicating that no structural changes have occurred during the ICD synthesis. The absorbance spectra of the IR780-I and [IR780][NOR] prepared in aqueous media show a slightly blue-shifted absorbance maximum wavelength at 776 nm as well as significantly reduced molar absorptivity (Figure 2a). This shift in absorbance wavelength and decrease in molar extinction coefficient is attributed to the inherent aggregation in the dispersed aqueous ICD (Jordan *et al*., 2012; m Chen *et al*., 2018). Interestingly, both aqueous solutions show an additional absorbance peak at 980 nm. This peak could allow activation of the drug by using electromagnetic radiation at longer wavelengths which is advantageous for PTT. Longer wavelength light has been demonstrated for superior penetration into the skin thus the added absorbance peak at 980 nm could lead to treating infections in deeper tissues (Finlayson *et al*., 2022).

Photophysical properties such as quantum yield, and radiative, and non-radiative rate constants are calculated using fluorescence emission spectra. The fluorescence emission spectra of IR780-I and [IR780][NOR] dissolved in DMSO showed identical fluorescence emission peaks at 907 nm when excited at 784 nm (Figure 2b). However, aqueous IR780-I and [IR780][NOR] both exhibited no fluorescence emission when excited at 784 nm. The absence of fluorescence emission is the first piece of evidence that the aqueous ICD will likely follow the non-radiative mechanism. Typically, after irradiation, the excited state molecules return to the ground state through the radiative pathway, as indicated by the fluorescence emission of the DMSO samples. However, in the case of the PTT mechanism, the excited state molecules ultimately follow the non-radiative relaxation pathway and thus no fluorescence emission signal can be observed (Konrad *et al*., 2016). For further confirmation of the presence of the PTT mechanism in an aqueous system, the relative quantum yield, and radiative and non-radiative rate constants were also calculated using Eq.s 1, 2, and 3 (Table 1). The relative quantum yield of ICD was calculated using IR780-I as the standard. The reported quantum yield value for IR780-I is 8.0% (Levitz *et al*., 2018).

As expected from the fluorescence emission spectra (Figure 2b) the quantum yields of the aqueous samples were significantly lower than that of the DMSO soluble samples. A decrease in the quantum yield quantifies a reduction in the fluorescence emission signal (Porres *et al*., 2006). A desirable photothermal agent must relax back to the ground state through the non-radiative pathway and thus generate heat. By inducing aggregation in the aqueous samples, the fluorescence pathway is no longer possible and allows the generation of more heat as an efficient photothermal agent (Liu *et al*., 2019). The changes in the K_r_ and K_nr_ are compared (Pochas, 2018). An approximately 10-fold increase in the K_nr_ is observed in both aqueous samples as compared to the DMSO soluble IR780-I and ICD, indicating that the PTT mechanism is enhanced in an aqueous environment.

The ICD must show high photostability due to the requirement of light to activate the PTT mechanism.

### Light to Heat Conversion Efficiency

**Figure 3.**
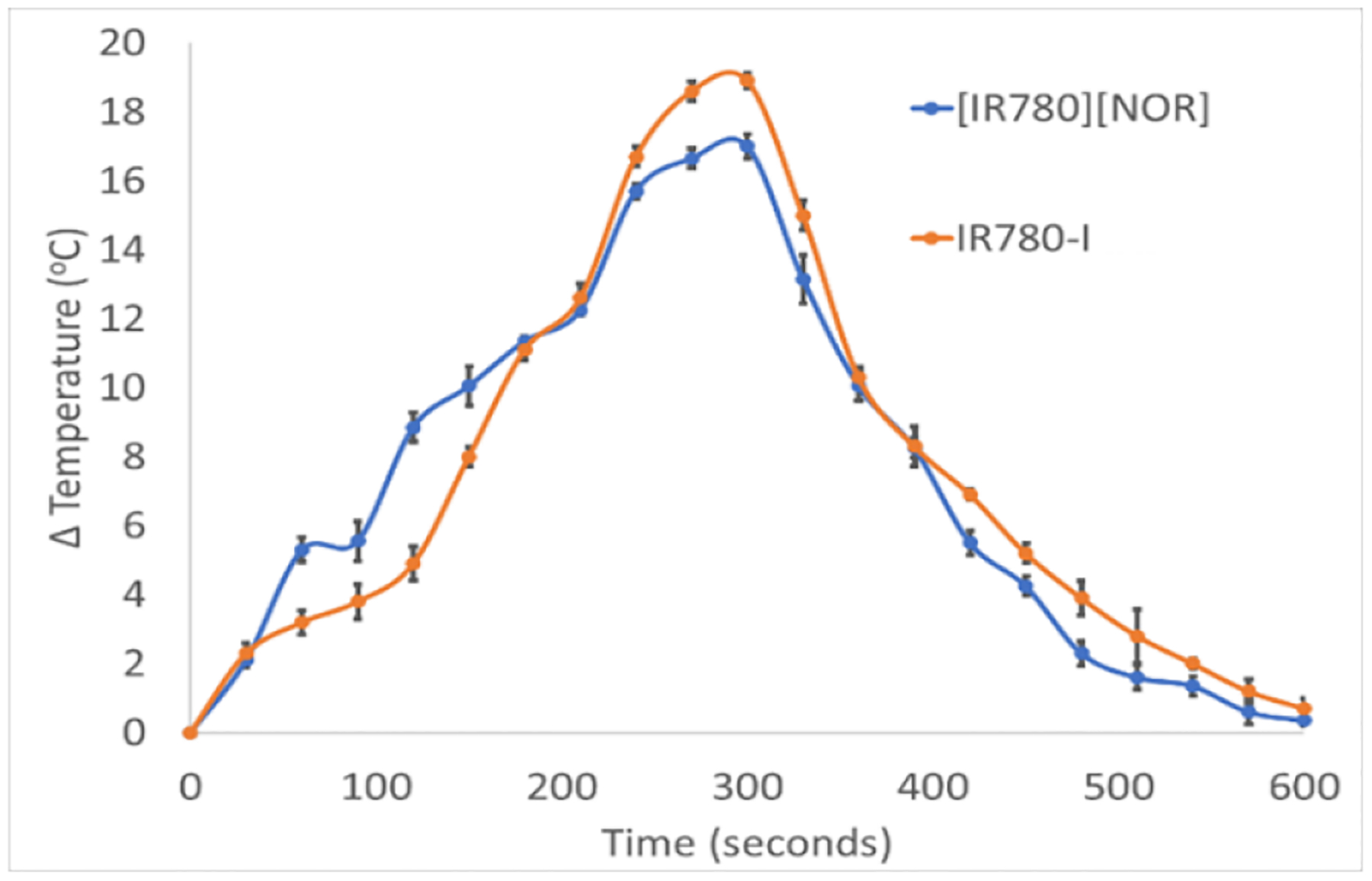
Heat efficiency charts for aqueous IR780-I (A) and [IR780][NOR] (B)

The promising non-radiative rate data directed us to design a light-to-heat conversion efficiency experiment that can validate their PTT activity. The potential use of the ICD as a photothermal agent was investigated by performing light-to-heat conversion efficiency experiments (Figure 3). Aqueous IR780-I and [IR780][NOR] show a significant change in temperature of 17.1 and 18.9 °C respectively, after 5-minute laser irradiation. The increase in temperature of the samples exceeds the required change of at least 12 °C to cause severe damage to bacteria cells, thus confirming the application of the ICD as a PTT agent (Qing *et al*., 2019). From this heating and cooling curve (Figure 3), the photon-to-heat transfer efficiency of IR780-I and [IR780][NOR] was calculated as 79.1% and 78.4%, respectively, using Eq.s 4, 5, 6, and 7. It demonstrated that IR780 retained the photothermal therapeutic potential when combined with NOR antibiotics.

### Bacterial Studies

#### Percent Inhibition Studies

**Figure 4.**
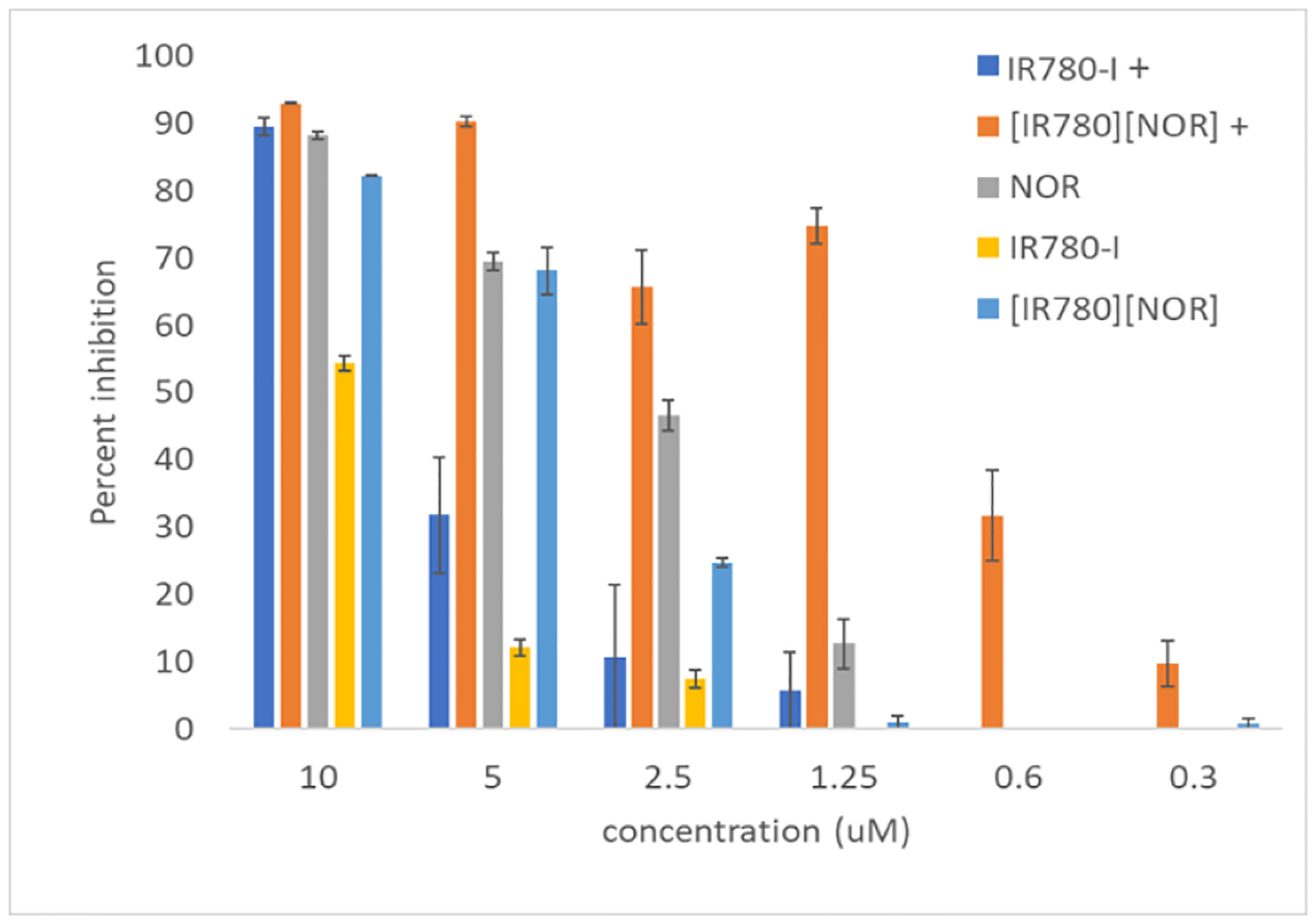
Percent inhibition of *E. coli* for each treatment. Samples denoted with ‘+’ were irradiated with an 808 nm laser for 5 minutes prior to incubation

Antibiotic activity of aqueous Na-NOR, IR780-I, and [IR780][NOR] were evaluated and compared by percent inhibition studies. The bacterial growth for each treatment was quantified relative to a positive and negative control. The percent inhibition was determined for each treatment at concentrations ranging from 10-0.3 μM (Figure 4). The antibiotic activity of each drug was investigated under dark conditions. The compounds containing the photoactive moiety, IR780, were irradiated with light and are denoted with a positive sign (IR780-I+ and [IR780][NOR]+). NOR does not absorb 808 nm light therefore it was not evaluated under irradiated conditions.

At the highest concentration (10 μM), all treatments exhibited the maximum antibiotic activity. Almost all drugs demonstrated more than 85% inhibition except the non-irradiated IR780-I sample which showed around 53% inhibition. The percent inhibition of the non-irradiated IR780-I sample was significantly lower than the NOR parent, ICD (non-irradiated and irradiated), and irradiated IR780-I (p=0.05) because there is no PTT mechanism in the absence of light. As the concentration of the drug increased, the non-irradiated IR780-I showed slight activity from 2.5 μM. The slight activity at higher concentrations is attributed to the chemical toxicity of the compound. In the non-irradiated IR780-I, the PTT mechanism is not active. PTT is the only killing mechanism present in IR780-I which can only be activated under light irradiation thus the non-irradiated IR780-I exhibited much lower activity than the other treatments. This lack of antibacterial activity reveals that the IR780-I is relatively non-toxic to bacteria without photoactivation.

When examining the activity of the PTT mechanism without the chemical killing mechanism, the irradiated IR780-I (IR780-I+) is considered. The irradiated IR780-I demonstrated comparable bacterial inhibition to the parent NOR and ICDs at the 10 μM concentration thus indicating the presence of the PTT mechanism. The PTT treatment alone is effective at high concentrations however a steep decline in the PTT activity is observed at lower concentrations. Under laser irradiation, IR780-I+ percent inhibition values declined as the concentration decreased and no PTT activity was observed after 0.6 μM. The waning of antibacterial activity at lower concentrations confirms that the PTT mechanism alone is not sufficient for bacterial killing.

The chemical killing mechanism was investigated by observing the parent NOR and non-irradiated [IR780][NOR]. The antibiotic activity of the parent NOR gradually declined as the drug concentration decreased until 0.6 μM. After 0.6 μM, no antibacterial activity is observed in the parent NOR samples. The non-irradiated [IR780][NOR] activity closely followed the parent NOR activity until 2.5 μM, at which point a significant difference in bacterial inhibition was observed (p=0.05). As previously concluded, IR780 does not provide the PTT mechanism in the absence of light irradiation. The [IR780][NOR] is not irradiated in this sample, therefore the antibiotic is the only active killing mechanism in this sample.

The effects of both mechanisms are assessed through the activity of the irradiated [IR780][NOR] ([IR780][NOR]+). The irradiated [IR780][NOR]+ demonstrated moderately high activity until the 0.6 μM concentration yet inhibition was observed. A slight decrease in [IR780][NOR]+ activity is noted at the 2.5 μM concentration but there is no significant difference when compared to the activity at the 5 and 1.25 μM concentrations (p=0.05). This enhanced antibacterial activity of [IR780][NOR]+ as compared to the parent NOR and non-irradiated [IR780][NOR] is attributed to the activation of the PTT mechanism upon irradiation. When comparing the two irradiated samples, the [IR780][NOR]+ showed substantially higher activity than the IR780-I+. The superior activity of the irradiated [IR780][NOR]+ as compared to the irradiated IR780-I+ indicates that the PTT mechanism is not solely responsible for the enhanced killing. Whereas, a synergistic effect of the chemical and physical killing mechanisms plays a role in attaining enhanced antibiotic activity even at lower concentrations of the irradiated [IR780][NOR]+ drug. It is possible that NOR^−^ is helping to reach bacteria and then the counterion, IR780^+^, starts generating heat under laser irradiation which aids in diminishing bacteria.

### Bacterial Uptake

**Figure 5.**
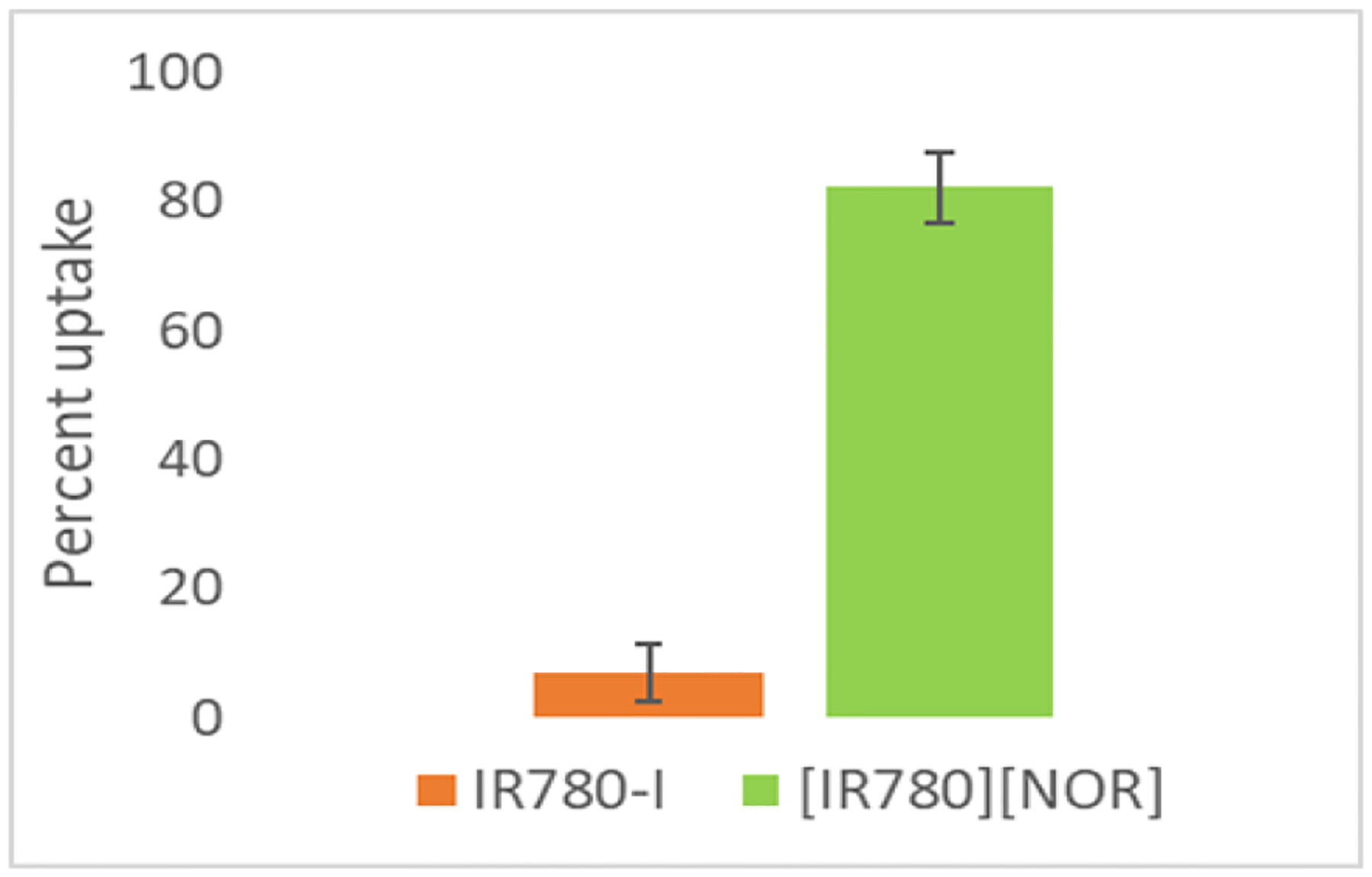
Percent uptake for IR780-I and [IR780][NOR]

To verify the enhanced activity of irradiated [IR780][NOR]+ as compared to IR780-I+, the cellular uptake experiment was designed. The PTT mechanism is present in both compounds when irradiated by an 808nm laser light. However, the irradiated IR780-I+ does not exhibit high activity toward the bacteria at lower concentrations than the ICD does. To explain this phenomenon, the bacterial uptake was evaluated (Figure 5). When the two moieties are combined in the ICD, the [IR780][NOR] shows superior bacterial uptake as compared to the IR780-I. The high uptake of ICD is attributed to the interaction of NOR with the bacterial cells. NOR binds with the replication complex in bacteria (Pham *et al*., 2019). This presence of NOR aids IR780 in the ICD to reach the bacteria and allows for a targeted and dual mechanism treatment.

## Conclusion

This novel ICD shows excellent potential as a combination therapy for antibacterial treatment. In the ICD, the IR780 moiety retains the photophysical properties of the parent IR780-I as observed by recording the absorbance and fluorescence emission spectra in organic and aqueous media. The light-to-heat transfer efficiency, a significant parament to evaluate the photothermal therapy efficiency of the drug, is consistent between the parent IR780-I and [IR780][NOR] as well. With the addition of the electrostatically joined NOR^−^ antibiotic with IR780^+^ in ICD, a synergistic effect is observed in the treatment of *E. coli* when irradiated with a laser of IR808 nm. The parent IR780-I shows poor antibacterial activity with and without irradiation where inhibition is only observed at the highest concentrations. The increased activity of [IR780][NOR] as compared to the parent IR780-I is attributed to the inclusion of the NOR moiety. NOR not only adds a chemical killing mechanism to the ICD but also increases the bacterial uptake significantly. [IR780][NOR] demonstrates higher antibiotic activity when irradiated as compared to the parent NOR, indicating that the PTT mechanism is contributing to bacterial killing. The combination of these two moieties has created a synergistic effect to efficiently kill *E. coli* cells at lower concentrations than that of the parent drugs. This study demonstrates that bacterial infection can be treated using lower concentrations of antibiotics after including the physical killing mechanism (in this case a PTT mechanism). This additional physical killing mechanism has tremendous potential to reduce the chances of resistance development. In addition, it can be a better strategy to treat resistant bacteria which cannot be treated by common antibiotics.

## Figures and Tables

**Table 1.** Molar extinction coefficient (ε), quantum yield (Φ), and radiative (k_r_) and non-radiative (k_nr_) rate constants of IR780-I (DMSO), [IR780][NOR] (DMSO), [IR780][NOR] (aq), and IR780-I (aq)

Compound	ε at 784 nm (×10^5^ M^−1^ cm^−1^)	Φ (%)	k_r_ (×10^7^ s^−1^)	k_nr_ (×10^8^ s^−1^)
IR780-I DMSO	1.81	8.0	1.21	1.39
[IR780][NOR] DMSO	1.62	9.0	1.12	1.14
[IR780][NOR] aq	1.22	0.8	1.41	17.8
IR780-I aq	1.20	0.7	1.34	19.2
